# The effects of electronic medical record phenotyping details on genetic association studies: HDL-C as a case study

**DOI:** 10.1186/s13040-015-0048-2

**Published:** 2015-05-06

**Authors:** Logan Dumitrescu, Robert Goodloe, Yukiko Bradford, Eric Farber-Eger, Jonathan Boston, Dana C Crawford

**Affiliations:** 1 Center for Human Genetics Research, Vanderbilt University, 2215 Garland Avenue, 519 Light Hall, Nashville, TN 37232 USA; 2 Department of Molecular Physiology and Biophysics, Vanderbilt University, 2215 Garland Avenue, 519 Light Hall, Nashville, TN 37232 USA; 3 Center for Systems Genomics, Department of Biochemistry and Molecular Biology, The Pennsylvania State University, 512 Wartik Laboratory, University Park, PA 16802 USA; 4 Department of Epidemiology and Biostatistics, Institute for Computational Biology, Case Western Reserve University, Wolstein Research Building, 2103 Cornell Road, Suite 2527, Cleveland, OH 44106 USA

**Keywords:** Electronic medical record, Genetic risk score, HDL-C, eMERGE network, PAGE I study

## Abstract

**Background:**

Biorepositories linked to de-identified electronic medical records (EMRs) have the potential to complement traditional epidemiologic studies in genotype-phenotype studies of complex human diseases and traits. A major challenge in meeting this potential is the use of EMR-derived data to extract phenotypes and covariates for genetic association studies. Unlike traditional epidemiologic data, EMR-derived data are collected for clinical care and are therefore highly variable across patients. The variability of clinical data coupled with the challenges associated with searching unstructured clinical notes requires the development of algorithms to extract phenotypes for analysis. Given the number of possible algorithms that could be developed for any one EMR-derived phenotype, we explored here the impact algorithm decision logic has on genetic association study results for a single quantitative trait, high density lipoprotein cholesterol (HDL-C).

**Results:**

We used five different algorithms to extract HDL-C from African American subjects genotyped on the Illumina Metabochip (n = 11,519) as part of Epidemiologic Architecture for Genes Linked to Environment (EAGLE). Tests of association between HDL-C and genetic risk scores for HDL-C associated variants suggest that the genetic effect size does not vary substantially across the five HDL-C definitions.

**Conclusions:**

These data collectively suggest that, at least for this quantitative trait, algorithm decision logic and phenotyping details do not appreciably impact genetic association study test statistics.

## Background

Biorepositories linked to de-identified electronic medical records (EMR) are an emerging resource for genetic association studies [1]. Compared with traditional epidemiologic studies, EMR-based studies offer multiple advantages including relative ease of ascertainment, rapid accrual of samples and associated data, longitudinal measures, and the potential for lengthy follow-up. Another major advantage of EMR-based or clinic-based studies is their potential for pharmaocogenomics and other applications associated with personalized medicine.

While clinic-based studies linked to EMRs offer multiple advantages, they also offer multiple challenges when accessed for research such as genetic association studies. A major challenge of the EMR is that the data are not collected for research purposes; that is, the data are collected as part of routine clinical care. Therefore, unlike traditional epidemiologic studies, there is no “baseline” measurement or examination of all study participants, and the number of overall measurements and exams can vary widely by patient. This variability is in stark contrast to longitudinal epidemiologic studies where participants are surveyed and examined uniformly every few years.

Because of the variable and somewhat erratic nature of the EMR data, investigators accessing these data for genetic association studies must make specific decisions in developing phenotype algorithms designed to extract outcomes and covariates for analysis. For example, for a commonly studied measurement such as body mass index, the investigator has multiple options including the first height and weight mentioned, the last height and weight mentioned, an average of all heights and weights mentioned for all clinic visits, the height and weight mentioned closest to another clinical diagnosis (such as type 2 diabetes), and so on.

Many of the challenges associated with EMR-based phenotyping are being addressed by collaborative consortiums such as the electronic MEdical Records and GEnomics (eMERGE) network, a cooperative group of several DNA biorepositories in the United States linked to EMRs funded by the National Human Genome Research Institute [2,3]. A major goal of the eMERGE network is the development of portable algorithms designed to define disease outcomes for use in genetic association studies [4]. Algorithms developed under eMERGE have been used successfully for single study site [5,6] and well as eMERGE-wide studies [7-13], the latter of which demonstrate the portability of these algorithms despite possible variations in clinical practice. A portion of the eMERGE EMR-derived phenotypes have also been mapped back to PhenX variables using the PhenX Toolkit [14], suggesting that EMR-derived phenotypes are comparable to epidemiologic collected phenotypes [15].

The eMERGE network has been successful in designing and implementing EMR-based algorithms for multiple phenotypes; however, it is unclear if the decision logic underlying each algorithm for phenotypes with repeated measures impacts downstream analyses for genetic association studies. To explore this possible impact, we as the Epidemiologic Architecture for Genes Linked to Environment (EAGLE) as part of the larger Population Architecture using Genomics and Epidemiology (PAGE) I study [16] conducted a genetic association study for the commonly measured and studied high density lipoprotein cholesterol (HDL-C). We created five HDL-C algorithms to extract this quantitative trait from African American subjects genotyped with the Illumina Metabochip [17] and available in BioVU, the Vanderbilt biorepository linked to de-identified electronic medical records [18]. Overall, we demonstrate that the genetic effect size estimates and levels of significance are similar across all HDL-C extraction methods attempted suggesting that commonly used decision logic for repeated measures in the EMR may not have appreciable impacts on downstream analyses conducted for genetic association studies.

## Methods

### Study population

All study subjects are drawn from BioVU, Vanderbilt University Medical Center’s biorespository linked to de-identified electronic medical records. A description of BioVU, including its oversight and ethics, has been previously published [18,19]. In brief, DNA is extracted from discarded blood samples drawn for routine clinical care from Vanderbilt University affiliated outpatient clinics. The DNA sample is linked to the patient’s de-identified EMR known as the Synthetic Derivative (SD). The SD contains billing (ICD-9) codes, procedure codes and labs. Prescription medication, including dose, is available in the SD through MedEx [20], an algorithm that extracts medications and their signature mentions from free-text entries available in the EMR. The SD also contains all clinical notes.

As EAGLE, a study site of PAGE I, we genotyped mostly non-European descent DNA samples available in BioVU as of 2011 on the Illumina Metabochip (described below), hereto referred as “EAGLE BioVU” (n = 15,863) [21]. The present study is limited to African Americans within EAGLE BioVU (n = 11,519).

### HDL-C definitions

HDL-C measurements were extracted from a de-identified EMR using five different methods. First, for each subject, the median HDL-C value of all documented HDL-C measurements was collected (“All HDL-C”). Next, both first and last reported HDL-C were mined from the subject’s laboratory data (“First HDL-C” and “Last HDL-C”). Lastly, HDL-C values were extracted for subjects both prior to (“pre-medication HDL-C”) or following (“post-medication HDL-C”) evidence of lipid-lowering medications and the median value was reported. EAGLE BioVU clinical notes were searched for evidence of lipid-lowering drugs for each subject using medication class as well as medication generic and brand names (Table 1). For each mention of lipid-lowering drug use, we extracted the date of medication mention to compare against date of HDL-C lab to determine if that measurement of HDL-C was “pre-medication” or “post-medication.” Subjects with no evidence of lipid-lowering medication prescriptions were considered “pre-medication HDL-C.” All HDL-C values used in this analysis were collected when the subject was 18 years or older.

**Table 1:** **Lipid**-**lowering medication class and list of drugs**

Fibrates	Resisns	Statins
Gemfibrozil (Lopid®)	Cholestyramine (Questran®, Questran® Light, Prevalite®, Locholest®, Locholest® Light)	Atorvastatin (Lipitor®)
Fenofibrate (Antara®, Lofibra®, Tricor®, Triglide™)	Colestipol (Colestid®)	Fluvastatin (Lescol®)
Clofibrate (Atromid-S)	Colesevelam Hcl (WelChol®)	Lovastatin (Mevacor® and Altoprev™)
		Pravastatin (Pravachol®)
		Rosuvastatin Calcium (Crestor®)
		Simvastatin (Zocor®)
		Lovastatin + niacin (Advicor®)
		Atorovastatin + amlodipine (Caduet®)
		Simvastatin + ezetimibe (Vytorin™)

Four major medication classes containing lipid-lowering medications were used to search the clinical notes: fibrates, niacin, resins, selective cholesterol absorption inhibitors (Ezetimibe or Zetia®), and statins (also known an HMG CoA reductase inhibitors). For each of the medication classes included in the search, we have listed the specific drugs considered, including both the generic and brand names.

### Genotyping and SNP selection

A total of 15,863 DNA samples from mostly non-European descent subjects were genotyped on the Illumina Metabochip, including 11,519 African Americans, by Vanderbilt University Center for Human Genetics Research DNA Resources Core. The Illumina Metabochip is a custom array of approximately 200,000 variants chosen as GWAS-identified index variants or GWAS-identified regions for fine-mapping based on data from the first iteration of the 1000 Genomes Project [17]. Quality control of the Illumina Metabochip data for EAGLE BioVU followed the quality control procedures outlined in Buyske et al. [22].

Based on a previous fine-mapping study of HDL-C using Metabochip [23], seven of the 22 fine-mapped HDL-C loci exhibited evidence of association at p < 1x10^−4^ in African Americans. The seven index SNPs from these seven associated HDL-C loci were selected for use in calculating the genetic risk score (GRS, Table 2).

**Table 2:** **SNPs used to calculate the genetic risk score for HDL**-**C in African Americans**

SNP	Gene of interest	Effect Allele	Effect on HDL-C^†^(mg/dl)
rs247617	*CETP*	C	−0.111
rs1077834	*LIPC*	A	−0.033
rs10096633	*LPL*	G	−0.042
rs189069311	*APOA5*	A	−0.080
rs255054	*LCAT*	A	−0.042
rs6601299	*PPP1R3B*	A	−0.063
rs4810479	*PLTP*	G	−0.029

^†^Beta coefficients were drawn from meta-analysis results of PAGE African Americans [23].

### Statistical methods

Both a weighted and unweighted GRS were calculated in PLINK [24]. In general, the GRS is calculated for each subject by counting the number of effect alleles (0, 1, or 2) across each SNP, multiplying that number by the known effect size (for the unweighted GRS, effect sizes were set equal to one), summing those values, and dividing by the number of non-missing SNPs, thus providing the average score per SNP. Effect estimates for the weighted GRS were based on the meta-analysis of PAGE African Americans [23]. Linear regression, adjusted for sex, with GRS as the independent variable and HDL-C measurement as the dependent variable was used to determine the beta coefficient.

## Results

Approximately 43% of the 11,519 African American subjects genotyped on the Illumina Metabochip as part of EAGLE had at least one HDL-C measurement available in the EMR (Table 3). The median number of clinic visits and medical records lengths in years was three each while the median ICD-9 code mentions (for unique codes) was 54. The median value for HDL-C ranged from 48–51 across the five different HDL-C definitions explored here (Table 3).

**Table 3:** **EAGLE BioVU African American demographics for HDL**-**C**

Variable	No. Obs.	Median	IQR
Medical record length (years)	4,912	3	8
Clinic visits (N)	4,912	3	6
ICD-9 codes (N)^†^	4,897	54	85
All HDL-C (mg/dl)	4,890	51	23
First HDL-C (mg/dl)	4,912	50	23
Last HDL-C (mg/dl)	4,912	49	22
pre-medication HDL-C (mg/dl)	4,074	51	23
post-medication HDL-C (mg/dl)	2,086	48	22

Number of observations (No. Obs.) as well as medians and interquartile ranges (IQR) are given for each variable. ^†^Includes only unique ICD-9 codes per individual.

**Figure 1:**
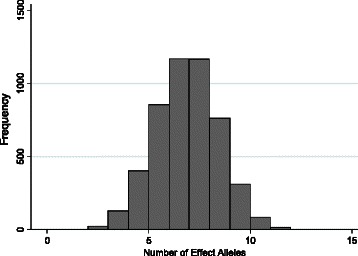
Distribution of HDL-C risk alleles in EAGLE BioVU African Americans.

We first calculated the unweighted GRS using seven HDL-C associated variants (Table 2) for each African American in EAGLE BioVU with at least one HDL-C measurement. The number of HDL-C risk alleles ranged from 3 to 12, which the majority of subjects having 8 risk alleles (Figure 1).

**Figure 2:**
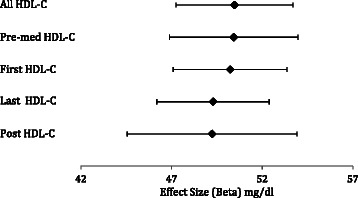
Additive effects of HDL-C risk alleles on various HDL-C measurements. Effect sizes (betas) from the linear regression analysis with the unweighted GRS, adjusted for sex, are shown as expected HDL-C levels (in mg/dl; black diamonds), along with their 95% confidence intervals.

We then performed tests of association for each of the five HDL-C definitions using the unweighted GRS as the independent variable. The unweighted GRS was significantly associated with each of the five HDL-C definitions, and the levels of significance ranged from 4.06 × 10^−86^ (post-medication HDL-C; n = 2,085) to 3.73 × 10^−197^ (first HDL-C; n = 4,910). Because level of significance is influenced by sample size, we then plotted each resulting beta and 95% confidence intervals to compare the effect sizes of the unweighted GRS across the five different HDL-C definitions (Figure 2). The unweighted GRS effect size was similar across the five different HDL-C definitions (Figure 2). Results from the weighted GRS do not appreciably differ from the unweighted results (data not shown).

## Discussion

We demonstrate here that for HDL-C, a commonly studied quantitative trait for cardiovascular disease risk, algorithm decision logic and phenotyping details applied to repeated measures available in the EMR do not appreciably affect downstream genetic association test statistics or overall study conclusions. These data, along with on-going algorithm development within the eMERGE network [5,25], suggest that phenotypes derived from EMR-based repositories are robust to the underlying variability inherent in clinical collections. Although not explicitly tested here, the similarities of genetic effect sizes observed here for the five HDL-C definitions in the same sample suggest that any one of these EMR-derived test statistics robust to algorithm decision logic can be included in meta-analyses with traditional epidemiologic studies.

While our data suggest that EMR-derived phenotypes may be robust to certain aspects of the algorithm decision logic and phenotyping details, these data do not imply that genetic association studies are not impacted by poor phenotyping. Substantial literature has documented the need for rigorous case/control phenotyping as misclassification of either can lead to loss of power [26,27]. Careful phenotyping can also lead to insights into biological mechanisms or disease processes [28,29]. Finally, careful phenotyping is also essential for creative study design and genetic discovery [30].

The present study focuses on examining the impact algorithm decision logic has on genetic associations related to a single quantitative trait, HDL-C. As such, the conclusions offered here may be limited to HDL-C or to quantitative traits defined from repeated measures available in the EMR. Further study is needed to more fully explore the limitations and impact algorithm decision logic may have on genetic association studies for binary clinical outcomes such as myocardial infarction or pharmacogenomic studies for traits such as warfarin dosing. For the HDL-C data included here, additional limitations of the present study include limitations associated with extracting HDL-C from the EMR. For example, we searched clinic notes for mentions of lipid-lowering medication classes and drugs (genetic and brand names), but we did not include any common misspellings of these search terms. It is possible, therefore, that the “pre-medication” HDL-C definition contains HDL-C measurements while the subject was on lipid-lowering medication. Another limitation of the EMR is that, unlike most epidemiologic studies, fasting status or time to last meal is not available as a structured field. Here, we assumed that the HDL-C measured in EAGLE BioVU was measured for subjects who fasted for at least eight hours. This assumption is most likely incorrect, but its violation is unlikely to impact HDL-C levels substantially.

Another limitation of the present study is related to sample size and power. We present here tests of association between various HDL-C derived variables and an unweighted GRS. The unweighted GRS, by design, is calculated by the number of risk alleles at loci known to be significantly associated with HDL-C levels. Therefore, with only a few thousand samples, we were able to statistically replicate the expected association between the unweighted GRS and the various HDL-C variables to further examine the genetic effect sizes estimated from these tests of associations. While the sample size of the present study was large enough for replicating known associations such as the loci represented in the unweighted GRS, the sample size is not large enough to perform discovery studies with the entire Metabochip dataset, even when limited to common variation (minor allele frequency >5%). Indeed, tests of association between the various HDL-C variables and common variants on the Metabochip failed to identify a statistically significant association after correction for multiple testing (data not shown). Furthermore, neither significance rankings nor genetic effect sizes could be reliably compared across HDL-C variables given the chance findings of non-significant tests of associations. Larger sample sizes are needed to make comprehensive comparisons of genetic effect sizes and significance rankings for EMR-derived phenotypes susceptible to algorithm decision logic and phenotyping details.

Despite the limitations, this study had multiple strengths including the depth of the clinical data and the diversity of EAGLE BioVU. EMR-derived datasets such as EAGLE BioVU coupled with genotype and sequence data promise to enrich existing and complimentary datasets for future genetic association studies for complex human diseases and traits.

## Conclusions

These data collectively suggest that, at least for HDL-C, algorithm decision logic and phenotyping details do not appreciably impact genetics association study tests statistics.

## References

[1] Manolio TA (2008). Biorepositories–at the bleeding edge. Int J Epidemiol.

[2] McCarty C, Chisholm R, Chute C, Kullo I, Jarvik G, Larson E (2011). The eMERGE network: a consortium of biorepositories linked to electronic medical records data for conducting genomic studies. BMC Med Genet.

[3] Gottesman O, Kuivaniemi H, Tromp G, Faucett WA, Li R, Manolio TA (2013). The Electronic Medical Records and Genomics (eMERGE) network: past, present, and future. Genet Med.

[4] Kho AN, Pacheco JA, Peissig PL, Rasmussen L, Newton KM, Weston N (2011). Electronic Medical Records for Genetic Research: Results of the eMERGE Consortium. Sci Transl Med.

[5] Ritchie MD, Denny JC, Crawford DC, Ramirez AH, Weiner JB, Pulley JM (2010). Robust replication of genotype-phenotype associations across multiple diseases in an electronic medical record. Am J Hum Genet.

[6] Denny JC, Ritchie MD, Crawford DC, Schildcrout JS, Ramirez AH, Pulley JM (2010). Identification of Genomic Predictors of Atrioventricular Conduction. Circulation.

[7] Denny JC, Crawford DC, Ritchie MD, Bielinski SJ, Basford MA, Bradford Y (2011). Variants near FOXE1 are associated with hypothyroidism and other thyroid conditions: using electronic medical records for genome- and phenome-wide studies. Am J Hum Genet.

[8] Turner SD, Berg RL, Linneman JG, Peissig PL, Crawford DC, Denny JC (2011). Knowledge-Driven Multi-Locus Analysis Reveals Gene-Gene Interactions Influencing HDL Cholesterol Level in Two Independent EMR-Linked Biobanks. PLoS One.

[9] Crosslin D, McDavid A, Weston N, Nelson S, Zheng X, Hart E (2012). Genetic variants associated with the white blood cell count in 13,923 subjects in the eMERGE Network. Hum Genet.

[10] Rasmussen-Torvik LJ, Pacheco JA, Wilke RA, Thompson WK, Ritchie MD, Kho AN (2012). High Density GWAS for LDL Cholesterol in African Americans Using Electronic Medical Records Reveals a Strong Protective Variant in APOE. Clin Trans Sci.

[11] Ritchie MD, Denny JC, Zuvich RL, Crawford DC, Schildcrout JS, Bastarache L (2013). Genome- and phenome-wide analyses of cardiac conduction identifies markers of arrhythmia risk. Circulation.

[12] Ding K, de Andrade M, Manolio TA, Crawford DC, Rasmussen-Torvik LJ, Ritchie MD (2013). Genetic Variants That Confer Resistance to Malaria Are Associated with Red Blood Cell Traits in African-Americans: An Electronic Medical Record-based Genome-Wide Association Study. G3: Genes|Genomes|Genetics.

[13] Crosslin DR, McDavid A, Weston N, Zheng X, Hart E, de Andrade M (2013). Genetic variation associated with circulating monocyte count in the eMERGE Network. Human Mol Gen.

[14] Hamilton CM, Strader LC, Pratt JG, Maiese D, Hendershot T, Kwok RK (2011). The PhenX Toolki: get the most from your measures. Am J Epidemiol.

[15] Pendergrass SA, Verma SS, Holzinger ER, Moore CB, Wallace J, Dudek SM et al. Next-generation analysis of cataracts: determining knowledge drive gene-gene interactions using Biofilter, and gene-environment interactions using the PhenX Toolkit. Pac Symp Biocomput 2013;147–158 PMC3615413 23424120

[16] Matise TC, Ambite JL, Buyske S, Carlson CS, Cole SA, Crawford DC (2011). The next PAGE in understanding complex traits: design for the analysis of population architecture using genetics and epidemiology (PAGE) study. Am J Epidemiol.

[17] Voight BF, Kang HM, Ding J, Palmer CD, Sidore C, Chines PS (2012). The metabochip, a custom genotyping array for genetic studies of metabolic, cardiovascular, and anthropometric traits. PLoS Genet.

[18] Roden DM, Pulley JM, Basford MA, Bernard GR, Clayton EW, Balser JR (2008). Development of a large-scale De-identified DNA biobank to enable personalized medicine. Clin Pharmacol Ther.

[19] Pulley J, Clayton E, Bernard GR, Roden DM, Masys DR (2010). Principles of human subjects protections applied in an Opt-Out, De-identified biobank. Clin Trans Sci.

[20] Xu H, Jiang M, Oetjens M, Bowton EA, Ramirez AH, Jeff JM (2011). Facilitating pharmacogenetic studies using electronic health records and natural-language processing: a case study of warfarin. J Am Med Inf Assoc.

[21] Crawford DC, Goodloe R, Farber-Eger E, Boston J, Pendergrass SA, Haines JL, et al. Leveraging epidemiologic and clinical collections for genomic studies of complex traits. Hum Hered. (in press). 10.1159/000381805 PMC4528966 26201699

[22] Buyske S, Wu Y, Carty CL, Cheng I, Assimes TL, Dumitrescu L (2012). Evaluation of the metabochip genotyping array in African Americans and implications for fine mapping of GWAS-identified loci: the PAGE study. PLoS One.

[23] Wu Y, Waite LL, Jackson AU, Sheu WHH, Buyske S, Absher D (2013). Trans-ethnic fine-mapping of lipid loci identifies population-specific signals and allelic heterogeneity that increases the trait variance explained. PLoS Genet.

[24] Purcell S, Neale B, Todd-Brown K, Thomas L, Ferreira MA, Bender D (2007). PLINK: a tool set for whole-genome association and population-based linkage analysis. Am J Hum Genet.

[25] McDavid A, Crane PK, Newton KM, Crosslin DR, McCormick W, Weston N (2013). Enhancing the Power of Genetic Association Studies through the Use of Silver Standard Cases Derived from Electronic Medical Records. PLoS One.

[26] Zondervan KT, Cardon LR (2007). Designing candidate gene and genome-wide case–control association studies. Nat Protocols.

[27] McCarthy MI, Abecasis GR, Cardon LR, Goldstein DB, Little J, Ioannidis JPA (2008). Genome-wide association studies for complex traits: consensus, uncertainty and challenges. Nat Rev Genet.

[28] Manolio TA (2013). Bringing genome-wide association findings into clinical use. Nat Rev Genet.

[29] Lees CW, Barrett JC, Parkes M, Satsangi J: New IBD genetics: common pathways with other diseases. Gut 2011. 10.1136/gut.2009.199679 21300624

[30] Plomin R, Haworth CMA, Davis OSP (2009). Common disorders are quantitative traits. Nat Rev Genet.

